# Creativity, serendipity, and collaboration: Cross‐cutting features of successful task‐sharing in comprehensive safe abortion care

**DOI:** 10.1002/ijgo.13011

**Published:** 2020-07-31

**Authors:** Annik M. Sorhaindo

**Affiliations:** ^1^ Independent Consultant in Reproductive and Sexual Health Mexico City Mexico

**Keywords:** Comprehensive safe abortion care, Country case study, Healthcare worker, Task‐sharing, WHO guideline

## Abstract

Limited capacity to deliver comprehensive safe abortion care and shortages in trained healthcare providers contribute to a lack of access to safe services. The World Health Organization published guidelines and recommendations on expanding health worker roles through task‐sharing as one way to address disparities. A multicountry case study was conducted in six diverse contexts (Bangladesh, Colombia, Ghana, Mexico City in Mexico, Sweden, and Tunisia) to determine the cross‐cutting strategies that enabled inclusion of a broader range of healthcare workers in comprehensive safe abortion care. Five strategies emerged: leveraging of favorable contexts, policies, and guidelines; use of evidence for advocacy; building upon existing task‐sharing; mitigation of negative responses to abortion and task‐sharing; and collaboration across sectors. The findings suggest that there are potential opportunities for stakeholders to employ these strategies in many contexts to broaden health worker roles in comprehensive safe abortion care.

## Synopsis

Existing programs suggest that task‐sharing in comprehensive safe abortion care is successfully implemented when stakeholders leverage favorable contexts, policies, and guidelines; collaborate across sectors; and effectively use evidence for advocacy.

## 1 Introduction

Lack of access to safe abortion services is a principal cause of unsafe abortion.^1^ Barriers to accessing safe services are created by legal restrictions, negative social and cultural beliefs and attitudes toward abortion, limited capacity to deliver comprehensive safe abortion care in health systems,^2^ and shortages in availability of trained healthcare providers.^3^, ^4^ Shortages of healthcare workers who can perform abortions may exist in the form of overall scarcity or subnational disparities; for example, in rural areas or in the public sector.^4^ These disparities are most prevalent among specialist physicians—the cadre of health professional that in many contexts is the primary or sole provider of safe abortion care.^3^, ^4^, ^5^

Broadening the cadres of skilled healthcare workers that can participate in the delivery of comprehensive safe abortion care improves access to abortion services.^5^ The World Health Organization (WHO) safe abortion guidelines state that abortion care can be delivered by any properly trained healthcare worker and can be safely delivered at the primary care and community level, appropriate to the type of service.^2^ As technologies for delivery of safe abortion care have advanced and simplified—particularly in the first trimester with use of vacuum aspiration and medical abortion—a wider range of nonphysican health workers (including nurses, midwives, and community health workers) have become increasingly involved in delivery of comprehensive safe abortion care.^3^, ^5^ Inclusion of a broader range of health workers in delivery of comprehensive safe abortion care addresses the problem of limited numbers of specialist physicians and reduces overall healthcare costs.^3^, ^5^

In July 2015, the WHO published guidelines including recommendations on expanding health worker roles in comprehensive safe abortion care through task‐sharing among a wider range of health workers.^5^ The evidence‐based guidelines outline the tasks and subtasks for which health workers beyond specialist physicians can safely and effectively deliver comprehensive safe abortion care. As part of the evidence base for development of the guidelines, researchers conducted case studies in five countries in which expansion of comprehensive safe abortion care to a broader range of health workers was already underway.^6^ The results of these case studies highlighted a series of observed barriers and facilitators, and the feasibility and acceptability of implementing task‐sharing in these five different countries. However, the case studies were limited by a dearth of documentation about the context and process of implementing task‐sharing in abortion service delivery. In many cases, countries simply did not document their process or the process was ad hoc. As a result, several gaps in evidence, knowledge, and understanding about the process of expansion of task‐sharing efforts persist. These gaps potentially thwart or delay in‐country implementation efforts. Further information on how to successfully implement task‐sharing in comprehensive safe abortion care in other national contexts can reduce repetition of unsuccessful strategies and encourage movement toward expansion of the comprehensive safe abortion care workforce and ultimately access to services.

To attempt to fill this gap, a further multicountry case study with a broader scope was designed and conducted to document the strategies that were undertaken to expand health worker roles in comprehensive safe abortion care in six diverse country contexts. The results of the individual case studies conducted in Bangladesh, Colombia, Ghana, Mexico City in Mexico, Sweden, and Tunisia were presented during a workshop at the fifth International Conference on Family Planning held in Kigali, Rwanda, in November 2018, and are reported in detail in this Supplement.^7^, ^8^, ^9^, ^10^, ^11^, ^12^

The present article considers the findings of the six case studies to assess the commonalities, differences, and potential universal lessons from the different contexts. The specific aim was to understand the features of the enabling contexts or strategies that facilitated successful inclusion of a broader range of healthcare workers in delivery of comprehensive safe abortion care. The analysis that follows distills these findings into fundamental lessons that can be applied elsewhere.

## 2 Materials and Methods

A multicountry case study was designed to illustrate the implementation of a policy intervention in a range of “real‐life” contexts. A case study design allows for intense study of a particular contemporary event or phenomenon, as a whole, in its naturally occurring context where behaviors or other actions are not manipulated.^13^ The approach is useful for in‐depth and multidimensional analysis of complex issues and provides the opportunity to understand processes, such as strategies undertaken to implement policy. Case studies rely on several sources of evidence for the purposes of the triangulation—or viewing evidence from different perspectives—to assure reliability of the findings.^13^

In this case study, six different countries that implemented a policy or practice to expand health worker roles in comprehensive safe abortion care were chosen: Bangladesh, Colombia, Ghana, Mexico City in Mexico, Sweden, and Tunisia. The countries represent diverse contexts of region, social and cultural environment, economic resources, healthcare system structure, abortion policy history and grounds for legal abortion, and length of experience of implementation. They were chosen because each has a program that formally includes nonspecialist physician health workers in delivery of comprehensive safe abortion care. As task‐sharing efforts increase, the specific political, economic, and cultural context of a country and its abortion legislation will determine the strategies necessary for implementing guidelines for task‐sharing in comprehensive safe abortion care in that specific context.

In each case study country setting, locally based researchers and experts systematically collected data and information from a range of sources to allow for triangulation of evidence and ultimately a holistic understanding of the process of implementation within each specific setting. Each country team collected data on:

- Comprehensive safe abortion care and task‐sharing policy.
- Relevant and available health information statistics.
- Published and grey literature.
- Interviews with key stakeholders.

The literature and interview data were analyzed separately for each country and are reported in the articles in this Supplement.^7^, ^8^, ^9^, ^10^, ^11^, ^12^ To identify facilitators and barriers to the implementation of policies to expand health worker roles in comprehensive safe abortion care, the principles of the SURE (Supporting the Use of Research Evidence) framework were used.^14^ The SURE framework offers a checklist of features of the policy implementation experience that have the potential to influence success.^14^ Using the data sources described above, similarities and differences across the countries were identified and are summarized in the present article.

## 3 Results

Exploration of the themes cutting across the six country contexts revealed five key strategies that appeared to facilitate the implementation of task‐sharing in comprehensive safe abortion care: (1) leveraging of favorable contexts, policies, and guidelines; (2) use of evidence as a mechanism for advocacy; (3) building upon existing task‐sharing in other areas of health care; (4) actively mitigating stigma and other negative responses to abortion and task‐sharing; and (5) collaboration involving a range of sectors.

### 3.1 Leveraging favorable contexts, policies, and guidelines

Across the six country contexts, stakeholders took advantage of existing circumstances to facilitate the inclusion of a broader range of health workers in comprehensive safe abortion care.

In Sweden, stakeholders capitalized on the existing positive perception of midwives and midwifery in Swedish society. Midwives in Sweden play a significant role in the delivery of healthcare services, especially sexual and reproductive health. Therefore, their participation in the delivery of comprehensive safe abortion was viewed by stakeholders, including physicians and women themselves, as a natural extension of their existing tasks. In fact, the skills developed in their existing roles, such as contraceptive counselling, lent themselves well to the delivery of medical abortion. Furthermore, interpretation of the law delegating tasks related to abortion allows for midwives to assume the bulk of the tasks associated with abortion care, while formally remaining a medical responsibility of physicians. The long‐standing respected position of midwives and the liberal interpretation of the law helped to facilitate their inclusion in comprehensive safe abortion care in Sweden.

In Bangladesh, menstrual regulation (use of manual vacuum aspiration or a combination of mifepristone and misoprostol to regulate the menstrual cycle when menstruation is absent for up to 10–12 weeks) has been permitted in the first trimester since 1979 and is typically delivered by nonphysican health workers. Stakeholders in Bangladesh did not resist the inclusion of nonphysican health workers in the delivery of comprehensive safe abortion care because they were already involved; for example, Family Welfare Visitors (FWVs) or Community Health Workers undertake a range of healthcare tasks and tend to focus on hard‐to‐reach and underserved populations, such as those living in remote, rural areas where there are fewer specialized physicians. Similar to the Swedish context, menstrual regulation was already accepted as a role that nonspecialist phyiscian health workers could naturally undertake, and their inclusion was viewed favorably.

Task‐sharing in sexual and reproductive health and other areas of health care in Colombia was already underway when abortion was decriminalized in 2006; nurses and nursing assistants are involved in prenatal care, contraception counselling, and sexually transmitted infection (STI) screening. Nurses in Colombia are also the cadre of health worker designated to attend to underserved and vulnerable populations. In addition, psychologists, social workers, and pharmacists are involved in health promotion and the delivery of health information. With this in mind, stakeholders in Colombia built upon the existing practice of task‐sharing in other healthcare services when preparing the guidelines and protocols for comprehensive safe abortion care. The Ministry of Health in Colombia produced guidelines stipulating that healthcare teams delivering comprehensive safe abortion care must include at least one nonspecialist physician, a counsellor, and a nurse. Nonspecialist physicians practicing in primary care facilities are required to be trained to provide medical abortion up to 10 weeks of gestation and manual vacuum aspiration (MVA) up to 15 weeks of gestation, and to identify and refer more complex cases.

In Ghana, the Ghana Health Service prepares nonphysician providers to support more highly trained health professionals, such as physicians, and step in when a more skilled health professional is unavailable. For example, health assistants are trained to support nurses, and medical assistants are trained to support physicians. Both health and medical assistants are equipped to provide care in the absence of the more highly trained provider. Additionally, nonphysician health workers, such as midwives, are taught to use MVA. Stakeholders and advocates for the inclusion of nonphysican health workers in comprehensive safe abortion care benefited from the precedent set by the Ghana Health Service and midwives’ existing training when nonphysican health workers were eventually included among the health workers permitted to deliver comprehensive safe abortion care in the country.

In Mexico City, clinical guidelines for comprehensive safe abortion care do not yet include nonphysican health workers, but the groundwork for future inclusion of nurses and midwives, to the extent that they are available, has been put in place. The national Ministry of Health has invested in the professionalization of nurses and increased federal funding to employ more nurses in a number of Mexican states. Additionally, legal and normative frameworks have included language that supports the involvement of trained nurses and traditional and professional midwives in attending low‐risk pregnancies and term births. In Mexico, comprehensive safe abortion care is not yet included in this framework, so this does not apply to procedures to terminate pregnancy. Nevertheless, the movement toward task‐sharing is underway and may include comprehensive safe abortion care in the future.

In all case study contexts, the availability of safe and simplified methods of safe abortion care, such as medical abortion and MVA, encouraged inclusion of a broader range of healthcare workers, as they could undertake the tasks with limited additional training. For example, in Tunisia, the legalization of safe abortion came in 1956 as part of a series of social and political changes following independence from France. Comprehensive safe abortion care was included as part of the national family planning program but designated as a service led by physicians. In the early 2000s when medical abortion regimens using mifepristone and misoprostol were introduced, midwives became involved in the delivery of services because it was viewed as a reasonable extension of their existing tasks.

### 3.2 Use of evidence as a mechanism for advocacy

Evidence was used in various ways to persuade stakeholders to include task‐sharing in comprehensive safe abortion care. In nearly all case study contexts, evidence was used to support the safety and effectiveness of a wider range of health workers in the delivery of safe abortion care. In several countries, local data specifically on feasibility and acceptability were leveraged to demonstrate the potential benefits of task‐sharing to providers and women.

Swedish researchers have produced a body of evidence on safe abortion, medical abortion, and task‐sharing in abortion care that has upheld the inclusion of midwives in comprehensive safe abortion care services in Sweden and contributed to the global evidence base on the safety, efficacy, feasibility, and acceptability of abortion. Recent research in Sweden has considered the simplification of safe abortion care, including self‐administration of medical abortion pills and self‐assessment of successful abortion.

In Ghana, evidence from a study designed to demonstrate the feasibility of midwife‐led postabortion care at the primary‐care level influenced the Ghana Health Service to reform their reproductive healthcare policy to allow providers with midwifery skills to deliver postabortion care. In both Ghana and Sweden, research and evidence on women’s perspectives were key in garnering support for the inclusion of midwives in the delivery of safe abortion care. Research in both countries found that women were satisfied with midwife‐provided care.

Stakeholders in Sweden and Colombia assessed evidence suggesting that task‐sharing in comprehensive safe abortion care with nonphysican health workers, particularly in the first trimester, reduced costs to women and health systems, and lowered waiting times. In Sweden, stakeholders acknowledged that midwife‐provided safe abortion care initially required resources to support training of midwives but, in the long run, midwife provision was more cost‐efficient than physician‐provided care.

Evidence may pave the way for Mexico City to include midwives in the delivery of safe abortion care. Following decriminalization of abortion in Mexico City, technical guidelines on the delivery of safe abortion care have become less medicalized. When the law was initially changed, the guideline stipulated that services had to be delivered in the hospital by specialized physicians. Current clinical guidelines have been loosened to allow services to take place in primary outpatient care facilities by general practitioners. Furthermore, with the inclusion of medical abortion, women are permitted to self‐administer misoprostol at home. Although general practitioners are responsible for determining gestational age, prescribing medical abortion medications, and conducting MVA, nurses are involved in preabortion counseling and provision of postprocedure family planning. However, the most recent effort to include nonphysican health workers into the cadres permitted to deliver safe abortion care in Mexico City failed in 2018.

Although it has not yet been applied to abortion care, international and national evidence was used in Mexico to support the development of an alliance between state and federal ministries of health throughout the country with bilateral organizations, such as UNFPA and the Pan American Health Organization (PAHO), academic institutions, professional associations, and midwives, among other key stakeholders, to develop a country‐specific model of midwifery that would broaden the cadre’s role in health care, including reproductive and maternal health, and potentially comprehensive safe abortion care.

A long history of research and numerous studies on the feasibility and efficacy of midwife‐led safe abortion care in Tunisia set the stage for health worker cadres to be permitted to participate in delivery of medical abortion in family planning clinics. Although physicians oversee the services and are responsible for prescribing medications, midwives are the main providers of the procedure and are involved in every stage of the process—from eligibility assessment through to prescribing postabortion contraception. Research in Tunisia has also produced evidence to support self‐administration of medical abortion and self‐assessment of abortion completion using urine pregnancy tests.

### 3.3 Building on existing task‐sharing in other areas of health care

As mentioned, in several of the case study contexts a broad spectrum of health workers was already involved in various aspects of health care—and in many contexts, reproductive health care—before they became involved in the delivery of comprehensive safe abortion care. The existing practice of training and involving a wide range of health workers in a variety of healthcare tasks typically designated for physicians appeared to ease the transition to task‐sharing in comprehensive safe abortion care.

Colombia and Bangladesh changed their policies on abortion decades apart. However, in both countries provisions for health workers beyond specialist physicians were written into safe abortion care delivery policy and guidelines from the inception of the programs. In Colombia, prior to the decriminalization of abortion, a number of tasks in sexual and reproductive health had already been distributed among different health workers. Beyond reproductive health, task‐sharing was already underway whereby nonphysician health workers participated in delivery of care for a range of conditions, such as tuberculosis and mental health. Abortion care advocates saw this as an opportunity when drafting the guidelines for comprehensive safe abortion care. Similarly, in Bangladesh nonphysican health workers such as nurses, paramedics, and FWVs have historically been involved in many aspects of health care and thus are also permitted to provide menstrual regulation up to 10 weeks of gestation.

Reproductive health policies and guidelines were reformed in 2003 in Ghana. The 2003 policy indicates that comprehensive abortion care can be delivered by trained health workers with midwifery skills, thereby allowing provision by a range of skilled health workers, including midwives and medical assistants. The guidelines for comprehensive safe abortion care, implemented in 2006, further permitted nurses with midwifery skills to perform first‐trimester abortion care.

Although Mexico City was the least progressive context for task‐sharing in comprehensive safe abortion care studied, the groundwork has been laid for inclusion of nurses in future safe abortion service delivery. In addition to a randomized controlled trial demonstrating that nurses are not inferior to doctors at delivering medical abortion,^15^ the General Health Law was modified to allow nurses to independently prescribe medications included in the country’s Essential Drugs List. Although there are limitations to what types of medications can be prescribed by nurses (which currently prevents them from prescribing the drugs used in medical abortion), there are likely to be future opportunities to broaden the scope of practice for nurses to include comprehensive safe abortion care.

In Tunisia, the Ministry of Health Office of Family Planning has historically supported task‐sharing and the involvement of midwives in a number of aspects of sexual and reproductive health service delivery, such as contraceptive counselling. In addition, the Office has promoted the involvement of midwives in comprehensive safe abortion care.

### 3.4 Actively mitigating stigma and other negative responses to abortion and task‐sharing

Inclusion of nonphysican health workers in comprehensive safe abortion care was often met with resistance from health workers themselves. However, stakeholders prioritized mitigating this tension and employed various tactics to do so. For example, in many contexts, improvements in and repetition of training, particularly values clarification, were used to encourage motivation and empowerment of nonphyiscan health workers and reduce negative sentiments.

In Colombia, task‐sharing in comprehensive safe abortion care grew out of task‐sharing in other areas of health; however, because abortion remains stigmatized in wider society and among health workers, the distribution of health workers performing abortion care services does not reflect what happens in task‐sharing elsewhere in the health system. However, technical and legal training for providers have appeared to improve attitudes toward abortion.

To combat resistance to task‐sharing in comprehensive safe abortion care in Ghana, stakeholders collaborated with a group of “champions,” including local government officials, representatives from women’s groups, and religious leaders, to address negative messages in the media about the program. There was also a mass media campaign to explain the new policy to providers in health facilities.

The Arab Spring in Tunisia increased conservatism and religious influence on society. As a result, providers are increasingly becoming stigmatized for offering comprehensive safe abortion care services. Advocates in Tunisia have dedicated considerable efforts to values clarification to ensure that task‐sharing does not lead to the stigmatization of cadres of health workers involved in delivering medical abortion. Furthermore, they are working to avoid task‐sharing becoming a mechanism for conservative providers to “off load“ the stigmatized safe abortion care service to other health worker cadres.

### 3.5 Collaboration involving a range of sectors

In all the case study contexts, implementation of task‐sharing was achieved through joint efforts across different agencies and sectors. The most common of these collaborations was between nongovernmental and civil society organizations and government agencies. Often, nongovernmental organizations (NGOs) have led the advocacy efforts with decision‐makers, political authorities, stakeholders, and medical and professional associations in sexual and reproductive health to encourage them to view task‐sharing as an efficient, safe, and valuable mechanism for broadening access to contraception and comprehensivesafe safe abortion care. Additionally, in nearly all the case study countries, international NGOs had a significant role in training cadres of healthcare workers to deliver family planning and comprehensive safe abortion care.

As one example of this, the international safe abortion organization, Ipas, worked closely with the Nursing and Midwifery Council in Ghana to promote the inclusion of midwives in comprehensive safe abortion care. In addition, it worked with the Ministry of Health to develop protocols and guidelines for the service and supported the design of the curriculum for training midwives in comprehensive safe abortion care.

NGOs and bilateral organizations, such as UNFPA, collaborated with government agencies in Bangladesh to train nonphysican health workers in the public and private sectors, and to add additional health workers, such as midwives, to menstrual regulation services.

The case study from Colombia highlights the implementation of task‐sharing in comprehensive safe abortion care from the perspective of a local NGO. In Colombia, stakeholders agreed that support from the Ministry of Health and Social Protection (MINSALUD) was key for implementing task‐sharing and the decentralization of comprehensive safe abortion care services. Collaboration with feminist collectives, such as the group La Mesa, and advocacy organizations eventually led to the decriminalization of abortion in the country and the articulation of guidelines and protocols favorable to task‐sharing and decentralization. It was also suggested that collaboration with health insurers could lead to further extension of task‐sharing in comprehensive safe abortion care services.

In most contexts, specialist physicians, namely obstetrician‐gynecologists, are the primary group of health professionals involved in the delivery of comprehensive safe abortion care. In Colombia and Sweden, collaboration with the obstetrics and gynecology associations—the Colombian Federation of Obstetrics and Gynecology (FECOLSOG) and the Swedish Society of Obstetrics and Gynecology (SFOG)—was important for including new cadres of health professionals into comprehensive safe abortion care. In Sweden, SFOG initiated the development of a curriculum to certify midwives in comprehensive safe abortion care in collaboration with the WHO Center at the Karolinska Institutet. Moreover, Colombian physicians report that they are less likely to be opposed to nurses’ involvement in abortion care if adequate training in ensured.^8^

In Mexico, an alliance between state and federal ministries of health and organizations such as UNFPA and PAHO, among others, has led to the development of a country‐specific model of midwifery that would broaden the cadre’s role in reproductive and maternal health.

## 4 Discussion

The six countries selected for the case studies were intentionally chosen to represent diverse contexts. Even though they are all quite different, there are some commonalities. In implementing task‐sharing in comprehensive safe abortion care in these diverse contexts, practitioners and key stakeholders looked for opportunities to “piggyback” comprehensive safe abortion care delivery on existing successful healthcare services, promoted task‐sharing in safe abortion care using evidence, and defended backlashes via training, values clarification, and advocacy campaigns. The strategies outlined in the present article can be utilized in new contexts to support the establishment of task‐sharing in abortion care.

As an initial step to understanding how national programs can bring their abortion services in line with the WHO recommendations, programs may conduct a systematic comparison similar to that done in India, published in this Supplement.^16^ This paper describes how stakeholders in India conducted an exercise to map existing comprehensive safe abortion care policy and guidelines within the country against the WHO guidelines. The exercise highlights the gaps and where investment is necessary to support expanding the number of cadres involved in the delivery of safe abortion care and access to services.

Task‐sharing can be an important part of ensuring access to comprehensive safe abortion care. Even in contexts that are currently restricted, efforts similar to those in Mexico City can be made to begin to lay the groundwork for inclusion of a broader range of health workers if and when the legal context changes. Furthermore, the WHO guidelines offer recommendations for inclusion of a broader group of health workers in postabortion care, which can be implemented in contexts were abortion is restricted.

Several of the case study locations (Bangladesh, Colombia, Ghana, and Mexico City) intended to broaden even further the health workers involved in comprehensive safe abortion care. These case studies should eventually be updated to document how national programs have succeeded in expanding their provider base for comprehensive safe abortion care.

Self‐administration of medical abortion, which is included in the WHO guidelines on task‐sharing, was not explicitly assessed in the case studies. Although women are permitted to administer some of the drugs at home in Mexico City, Sweden, and Tunisia, the case studies did not directly explore women’s role in and experiences with comprehensive safe abortion care. Future research should document how countries achieved decentralization of medical abortion administration by involving women in their own care.

## Author contributions

AS designed and coordinated the case studies including data collection.

## Conflicts of interest

The author has no conflicts of interest.

## References

[1] Ganatra B , Gerdts C , Rossier C , et al. Global, regional, and subregional classification of abortions by safety, 2010–14: Estimates from a Bayesian hierarchical model. Lancet. 2017;390:2372–2381. 28964589 10.1016/S0140-6736(17)31794-4 PMC5711001

[2] World Health Organization . Safe Abortion: Technical and Policy Guidance for Health Systems, 2nd edn. Geneva: WHO; 2012. 23700650

[3] Berer M . Provision of abortion by mid‐level providers: International policy, practice and perspectives. Bull World health Organ. 2009;87:58–63. 19197405 10.2471/BLT.07.050138 PMC2649591

[4] Campbell J , Dussault G , Buchan J , et al. A Universal Truth: No Health Without a Workforce. Forum Report, Third Global Forum on Human Resources for Health, Recife, Brazil. Geneva: Global Health Workforce Alliance and World Health Organization; 2013.

[5] World Health Organization . Health Worker Roles in Providing Safe Abortion Care and Post‐abortion Contraception. Geneva: WHO; 2015. 26401543

[6] Glenton C , Sorhaindo AM , Ganatra B , Lewin S . Implementation considerations when expanding health worker roles to include safe abortion care: A five‐country case study synthesis. BMC Public Health. 2017;17:730. 28934942 10.1186/s12889-017-4764-z PMC5609023

[7] Sultana N . Task‐sharing in menstrual regulation services: Implementation efforts and lessons learned in Bangladesh. Int J Gynecol Obstet. 2020;150(Suppl 1):4–8. 10.1002/ijgo.13009 PMC7540356 33219997

[8] Vivas MM , Valencia S . The extent of task‐sharing implementation as a strategy to expand abortion services in Colombia. Int J Gynecol Obstet. 2020;150(Suppl 1):9–16. 10.1002/ijgo.12999 PMC7540340 33219996

[9] Aborigo RA , Moyer CA , Sekwo E , et al. Optimizing task‐sharing in abortion care in Ghana: Stakeholder perspectives . Int J Gynecol Obstet. 2020;150(Suppl 1):17–24. 10.1002/ijgo.13000 PMC7540373 33219998

[10] Schiavon R , Troncoso E . Inequalities in access to and quality of abortion services in Mexico: Can task‐sharing be an opportunity to increase legal and safe abortion care? Int J Gynecol Obstet. 2020;150(Suppl 1):25–33. 10.1002/ijgo.13002 PMC7540305 33219993

[11] Endler M , Cleeve A , Sääv I , Genzell‐Danielsson K . How task‐sharing in abortion care became the norm in Sweden: A case study of historic and current determinants and events. Int J Gynecol Obstet. 2020;150(Suppl 1):34–42. 10.1002/ijgo.13003 PMC7539959 33219992

[12] Hajri S , Belhadj H . The role of midwives in first‐trimester abortion care: A 40‐year experience in Tunisia. Int J Gynecol Obstet. 2020;150(Suppl 1):43–48. 10.1002/ijgo.13010 PMC7539995 33219991

[13] Yin RK . Case Study Research. Design and Methods. 4th edn. Thousand Oaks, CA: Sage Inc; 2009.

[14] SURE . SURE Guides for Preparing and Using Evidence‐Based Policy Briefs: 5. Identifying and addressing barriers to implementing policy options. Version 2.1 [updated November 2011]. The SURE Collaboration 2011.

[15] Diaz Olavarrieta C , Ganatra B , Sorhaindo A , et al. Can nurses offer early medical abortion as safely and effectively as physicians? A randomized controlled non‐inferiority trial in Mexico City public legal abortion facilities, 2015. WHO Bulletin; doi: 10.2471/BLT.14.143990. 10.2471/BLT.14.143990 PMC4431559 26229189

[16] Manning V , Ganatra B , Gandhi M , Patil A . Adapting the WHO recommendations on health worker roles for safe abortion to a country setting: A case study from India. Int J Gynecol Obstet. 2020;150(Suppl 1):55–64. 10.1002/ijgo.13001 PMC7540050 33219994

